# VEGFR2 heterogeneity and response to anti-angiogenic low dose metronomic cyclophosphamide treatment

**DOI:** 10.1186/1471-2407-10-683

**Published:** 2010-12-15

**Authors:** Steven G Patten, Una Adamcic, Kristen Lacombe, Kanwal Minhas, Karolina Skowronski, Brenda L Coomber

**Affiliations:** 1 Department of Biomedical Sciences, Ontario Veterinary College, University of Guelph, Guelph, ON Canada N1G 2W1

## Abstract

**Background:**

Targeting tumor vasculature is a strategy with great promise in the treatment of many cancers. However, anti-angiogenic reagents that target VEGF/VEGFR2 signaling have met with variable results clinically. Among the possible reasons for this may be heterogeneous expression of the target protein.

**Methods:**

Double immunofluorescent staining was performed on formalin-fixed paraffin embedded sections of treated and control SW480 (colorectal) and WM239 (melanoma) xenografts, and tissue microarrays of human colorectal carcinoma and melanoma. Xenografts were developed using RAG1^-/- ^mice by injection with WM239 or SW480 cells and mice were treated with 20 mg/kg/day of cyclophosphamide in their drinking water for up to 18 days. Treated and control tissues were characterized by double immunofluorescence using the mural cell marker α-SMA and CD31, while the ratio of desmin/CD31 was also determined by western blot. Hypoxia in treated and control tissues were quantified using both western blotting for HIF-1α and immunohistochemistry of CA-IX.

**Results:**

VEGFR2 is heterogeneously expressed in tumor vasculature in both malignant melanoma and colorectal carcinoma. We observed a significant decrease in microvascular density (MVD) in response to low dose metronomic cyclophosphamide chemotherapy in both malignant melanoma (with higher proportion VEGFR2 positive blood vessels; 93%) and colorectal carcinoma (with lower proportion VEGFR2 positive blood vessels; 60%) xenografts. This reduction in MVD occurred in the absence of a significant anti-tumor effect. We also observed less hypoxia in treated melanoma xenografts, despite successful anti-angiogenic blockade, but no change in hypoxia of colorectal xenografts, suggesting that decreases in tumor hypoxia reflect a complex relationship with vascular density. Based on α-SMA staining and the ratio of desmin to CD31 expression as markers of tumor blood vessel functionality, we found evidence for increased stabilization of colorectal microvessels, but no such change in melanoma vessels.

**Conclusions:**

Overall, our study suggests that while heterogeneous expression of VEGFR2 is a feature of human tumors, it may not affect response to low dose metronomic cyclophosphamide treatment and possibly other anti-angiogenic approaches. It remains to be seen whether this heterogeneity is partly responsible for the variable clinical success seen to date with targeted anti-VEGFR2 therapy.

## Background

Solid tumors rely on a robust vascular supply for growth and spread to distant sites, thus blocking tumor growth by disrupting angiogenesis is a rational anti-cancer strategy and several targeted therapies are currently approved or are in clinical trial [1]. Conventional cytotoxic chemotherapy is administered at maximum tolerated doses (MTD) every 2-3 weeks, but when the same agents are administered on a low does metronomic (LDM) schedule (*i.e*. more frequently and at 10 fold or lower concentration), an anti-angiogenic effect occurs [2]. For example, vinblastine at ultra low doses (≤ 1 pmol/L) inhibits endothelial cell proliferation, migration and metalloproteinase secretion *in vitro *and *in vivo*. Paclitaxel, cyclophosphamide, and docetaxel exhibit similar effects on endothelial cells when given as LDM treatments [3,4]. Low dose metronomic cyclophosphamide has been shown to exert its effect through the induction of thrombospondin-1 (TSP-1), which presumably exerts its anti-angiogenic effects via binding to the receptor CD36 on the surface of endothelial cells [5]. TSP-1 can also bind and sequester VEGF, effectively inhibiting its pro-angiogenic effects [6]. VEGF activation of VEGFR2 (*KDR; flk-1*) is considered to be the major signal transduction event leading to both physiological and pathological angiogenesis [7].

Tumor blood vessels are structurally and functionally abnormal, resulting in relatively inefficient tissue perfusion despite high vascular density [8,9]. Administration of anti-angiogenic drugs should induce a reduction in tumor vessel density, due to destruction of unstable vessels and/or prevention of new sprouting. Anti-angiogenic agents may also generate a normalization window, where the tumor vasculature reverts to a more regular and organized state characterized by increased tumor oxygenation, improved drug penetration, and decreased interstitial fluid pressure[10-13]. While LDM cyclophosphamide did not induce significant blood vessel normalization in the RIP1-Tag2 pancreatic insulinoma model [14], it is not known whether vessels recruited to xenografted human cancer will be normalized by this therapeutic approach.

Previous work in our laboratory reported heterogeneous vascular expression of the Tie2/TEK receptor tyrosine kinase in some xenografted tumors, which were refractive to Tie2 inhibition[15]. Since variation in other receptor molecules could impact the effectiveness of anti-angiogenic agents, here we evaluate patterns of VEGFR2 expression by tumor vasculature, and explore the therapeutic impact of heterogeneous VEGFR2 expression during response to low dose metronomic cyclophosphamide using colorectal cancer and malignant melanoma tumors.

## Methods

### Cell Lines

Human malignant melanoma (WM239) and colorectal carcinoma (SW480) cell lines were obtained from the American Type Culture Collection (Manassas, VA, USA). Cells were cultured in DMEM (Sigma-Aldrich, Oakville, ON, Canada) containing 10% fetal bovine serum (FBS) (Invitrogen, Burlington, ON, Canada), 1% sodium pyruvate (Sigma-Aldrich), and 0.5% gentamicin (Invitrogen) and maintained in a humidified atmosphere at 37°C and 5% CO_2_.

### Xenografts

All animal studies were performed according to regulations of the Canadian Council on Animal Care as supervised by the local Animal Care Committee of the University of Guelph. Xenografts were established in male and female RAG1^-/- ^mice by injecting 2 × 10^6 ^WM239 or SW480 cells in 100 μL of 0.5% BSA/PBS subcutaneously into the right flank. Tumor size was monitored through caliper measurements taken every third day, and volume calculated using the formula (length × width^2^)/2. Tumors were allowed to grow to approximately 200 mm^3^, and then mice were randomized into treatment and control groups. Treatment consisted of 20 mg/kg/day of cyclophosphamide monohydrate (Sigma-Aldrich) in their drinking water[16]. Water for all mice was changed twice weekly for a period of up to 18 days, mice were euthanized by CO_2 _asphyxiation and cervical dislocation, and tissue samples were collected for analysis.

### Blood Vessel Assessment

Formalin-fixed paraffin embedded sections of treated and control SW480 and WM239 xenografts, and tissue microarrays of human colorectal carcinoma (Tissue Array Research Program, Centre for Cancer Research, NCI, Frederick, MD, USA), and melanoma (Imgenex, San Diego, CA, USA) were de-paraffinized and subjected to a 10 mM sodium citrate buffer, pH 6.0 for antigen retrieval. Sections were then incubated in DAKO Protein Block (DAKO, Mississauga, ON, Canada) for 1 hour. Antigen detection was performed sequentially by first using goat polyclonal anti-CD31 primary antibody (1:100; Santa Cruz Biotechnology, Santa Cruz, CA, USA) then donkey anti-goat FITC-conjugated secondary antibody (1:200; Santa Cruz Biotechnology) for 30 minutes. Sections were washed and then incubated with rabbit anti-VEGFR2 (1:100; Cell Signaling, Boston, MA, USA) overnight at 4°C, followed by goat anti-rabbit Cy3 (1:200; Jackson ImmunoResearch, West Grove, PA, USA) for 30 minutes. Antigen detection was performed sequentially (as described above). For mural cell quantification, antigen retrieval was in Tris-EDTA buffer solution (pH 9.0), followed by DAKO Protein Block and 5% donkey serum each for 30 minutes. Sections were incubated with goat polyclonal anti-CD31 (1:100; Santa Cruz Biotechnology) for 1 hour then donkey anti-goat FITC-conjugated (1:200; Santa Cruz Biotechnology) for 30 minutes, followed by anti-smooth muscle actin, directly conjugated to Cy3 (1:400; Sigma-Aldrich) for 30 minutes.

Five random fields of view at 200X magnification for each xenograft specimen were captured in a blinded fashion using QCapture software calibrated to a Leica DMLB microscope with an attached Q imaging QICAM fast1394 digital camera. One image/spot at 200X magnification was captured from each specimen in human tissue microarrays. Images were overlaid using Adobe Photoshop 7.0 (Adobe, Toronto, ON, Canada). VEGFR2 positive and negative, anti-smooth muscle actin positive and negative, and total blood vessels per field were quantified from overlaid images as previously reported[15]. Briefly, positive blood vessels were characterized by staining for both VEGFR2 or anti-smooth muscle actin antibodies, and the pan-endothelial cell marker, CD31. Vessel profiles with discontinuous CD31 staining were counted as separate vessels. Branched vessels were counted as a single vessel as long as staining for CD31 and/or VEGFR2 was continuous for the extent of the visible blood vessel profile. Microvessel density was calculated by dividing the total number of blood vessels per field area to determine the number of blood vessels per mm^2^.

### Immunohistochemistry for Hypoxia

Formalin-fixed paraffin embedded sections were de-paraffinized, rehydrated and incubated in 3% H_2_O_2 _(Fisher, Ottawa, ON, Canada) for 15 minutes, followed by antigen retrieval with acidic citrate buffer as described above. Sections were incubated in DAKO Protein Block for 20 minutes followed by 5% normal goat serum (Vector, Burlington, ON, Canada) for 45 minutes and rabbit anti-carbonic anhydrase-IX (1:500; Abcam, Cambridge, MA, USA) overnight at 4°C. Slides were washed, incubated in biotinylated goat anti-rabbit (1:500; Vector) for 30 minutes then treated with RTU Vectastain Elite, ABC reagent (Vector) for 30 minutes followed by AEC chromogen (Vector) for 5 minutes. Sections were counterstained with Mayer's hematoxylin (Sigma-Aldrich). Relative hypoxic tissue (as CA-IX positive regions) to total section surface area was calculated using the software ImageScope (Aperio, Vista, CA, USA). Sections were evaluated in a blinded fashion.

### Western Blotting

Tissues were lysed with Cell Lysis Buffer (Cell Signaling Technology) with added aprotinin, PMSF and Phosphatase Inhibitor Cocktail II (all from Sigma-Aldrich). SDS-PAGE was performed with 90 micrograms of total protein loaded into 7.5% polyacrylamide gels and proteins were transferred to PVDF membranes (Roche) and blocked in 5% milk/TBST. Membranes were probed with the following primary antibodies overnight at 4ºC: rabbit anti-VEGFR2 (1:1000; Cell Signaling Technology), mouse anti-TSP-1 (1:500; Lab Vision), mouse anti-tubulin (1:200,000; Sigma-Aldrich), mouse anti-HIF1α (1:400, R&D Systems, Minneapolis, MN), rabbit anti-desmin (1:1500; Abcam), goat anti-CD31 (1:800; Santa Cruz Biotechnology), followed by secondary antibodies goat anti-rabbit POD (1:5000 - 1:20,000), goat anti-mouse POD (1:20,000) or rabbit anti-goat (1:20,000, all from Sigma-Aldrich) for 30 minutes at room temperature. Protein bands of interest were detected with Chemiluminescence Blotting Substrate (Roche), and densitometry performed by normalization to tubulin signal. For desmin blots, signal was normalized to CD31 for each sample.

### *In Vitro *Cyclophosphamide Toxicity

Briefly, 1 × 10^4 ^cells were plated into each well in a 96 well plate in complete medium for 48 hrs then serum starved for 24 hrs. Plates were then exposed to 0-100,000 ng/ml 4-HC (4-hydroperoxycyclophosphamide; HCNiomech/IIT GmbH, Bielefeld, DE; the precursor of 4-hydroxycyclophosphamide, the metabolically active metabolite of cyclophosphamide) and incubated for 72 hours. MTT cytotoxicity assay was performed with a Cell Growth Determination Kit using the manufacturer's protocol (Sigma-Aldrich, Oakville, ON) and absorbance was read at 570 nm with an EL_X_800 Universal Microplate Reader (BIO-TEK Instruments Inc, Winooski, VT). Three independent replicates were preformed with each dose done in triplicate.

### VEGF-A ELISA

WM239 and SW480 cells were seeded into 6-well plates at a density of 5.0 × 10^5 ^cells. The following day, media was changed to DMEM and 2% FBS. 24 hours later, media was replaced to DMEM and 2% FBS ± CoCl_2 _(100 μM), ± 4-HC (100 nM) or a combination of both. 24 hours after treatment, condition media was collected and cell free supernatant was used to measure VEGF-A levels with ELISA using a commercially available ELISA kit (R & D Systems). Cells were counted to standardize VEGF levels. Two independent experiments were performed, and each sample analyzed as duplicates. Results were presented as pg VEGF per 10,000 cells.

### Quantification of TUNEL Reaction

Apoptosis was detected in paraffin embedded tissues with the *In Situ *Cell Death Detection Kit, POD (Roche), according to manufacturer's instructions. TUNEL-positive nuclei were identified with DAB substrate chromogen system (DAKO) and counterstained with Mayer's hematoxylin (Sigma-Aldrich). Quantification was performed in a blinded fashion by scoring the number of positive nuclei/10 high power fields of non-necrotic tissue. Five sections per group were analyzed and averaged.

### Statistical analysis

Comparison of means was performed using either the Student's *t*-test, or ANOVA followed by post-hoc analysis using the Bonferroni and Tukey's LSD methods. Differences were considered significant when p ≤ 0.05. Data are expressed as the mean and standard error of the mean (SEM).

## Results

### VEGFR2 Status of Blood Vessels in Human Tumors

**Figure 1:**
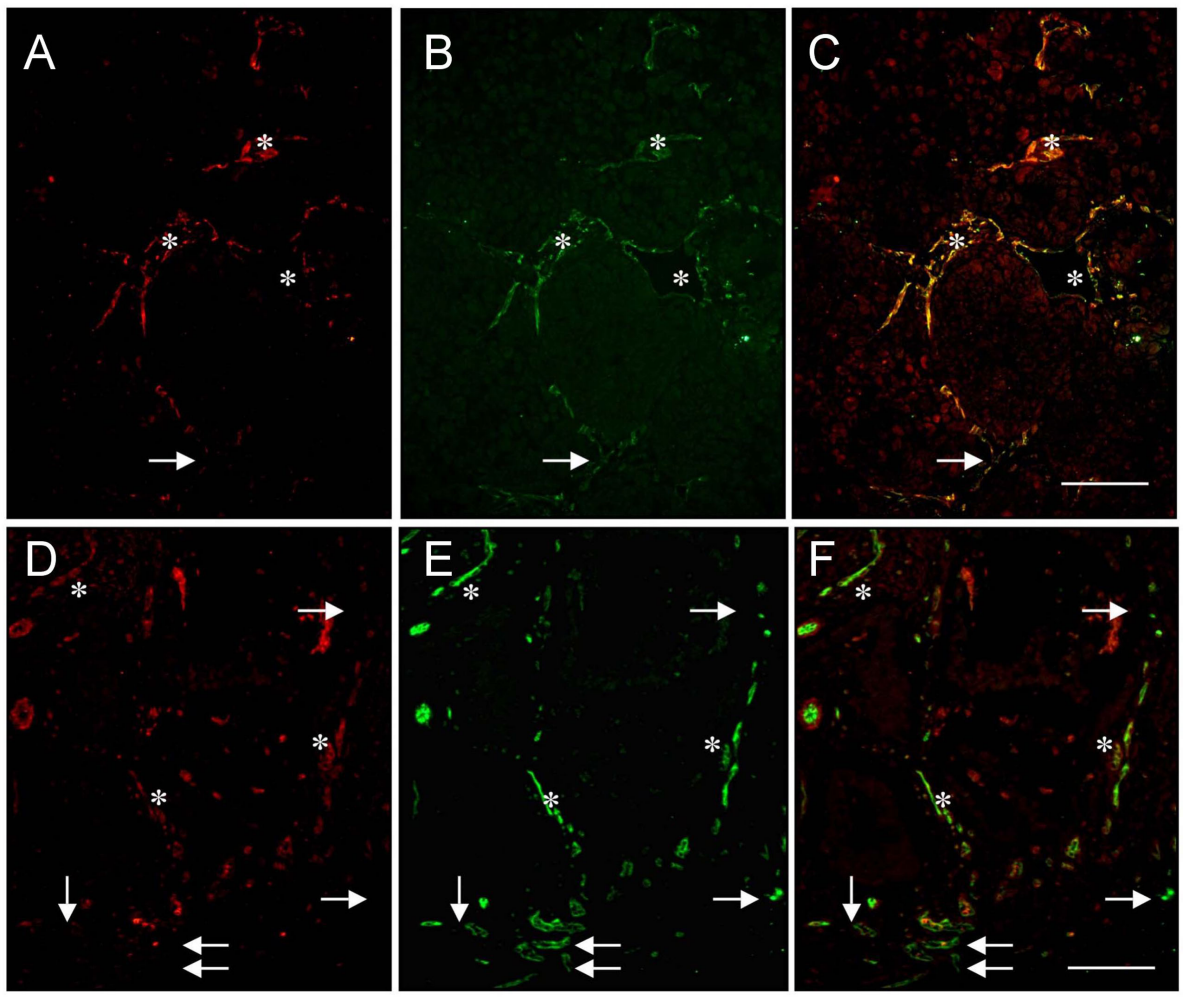
**Heterogeneous expression of VEGFR2 in melanoma and colorectal cancer**. Dual immunofluorescent staining for VEGFR2 (red) and CD31 (green) in human cancer specimens shows VEGFR2 negative blood vessels (arrows) and VEGFR2 positive blood vessels (asterisks). (A-C) Metastatic malignant melanoma and (D-F) colorectal carcinoma have been divided into their respective channels (overlaid; green; red images). Scale bar = 50 μm.

Dual immunofluorescence staining for VEGFR2 and CD31 was performed on formalin-fixed paraffin embedded tissue microarrays to determine the heterogeneous nature of VEGFR2 expression within the vasculature of clinically relevant tumors. Colorectal carcinoma (n = 36; on average approximately 20 blood vessels/tissue core), and metastatic malignant melanoma (n = 14; approximately 15 blood vessels/tissue core) were evaluated. Heterogeneous expression of VEGFR2 was observed in both tumor types (Figure 1; Table 1). Colorectal carcinoma had a significantly lower average percent VEGFR2 positive blood vessels (60% VEGFR2 positive blood vessels) than malignant melanoma (93% VEGFR2 positive blood vessels) (p < 0.05).

**Table 1:** Patterns of endothelial VEGFR2 expression in tumor blood vessels in xenografted tumors and clinical cancer

Tumor Type	# of Tumors	% VEGFR2 Positive	% VEGFR2 Negative
SW480 xenograft	4	60 ± 10.57^a^	40 ± 12.61^c^

Clinical colorectal carcinoma	36	60 ± 3.06^a^	40 ± 2.54^c^

WM239 xenograft	10	91.96 ± 3.01^b^	8.04 ± 0.41^d^

Clinical metastatic melanoma	14	93 ± 3.29^b^	7 ± 0.33^d^

**Table I - **VEGFR2 heterogeneity was assessed in human clinical specimens and xenografts of both colorectal carcinoma and metastatic malignant melanoma using dual immunofluorescence staining for VEGFR2 and CD31. The mean values for average percentage of VEGFR2 positive and VEGFR2 negative vessels were calculated along with SEM. Values with the same superscript (a-m) are significantly different from the same vessel class in different cancer types, but not from each other (p < 0.05).

### Conserved VEGFR2 Expression Patterns between Xenograft and Human Clinical Tumors

The VEGFR2 status of vessels in colorectal carcinoma and melanoma was compared between human clinical samples and mice xenografted with human cancer cells of the same type, and there were no significant differences in vascular phenotype between xenografts and clinical cases within a cancer type (Table 1) (p < 0.05). We consistently found that colorectal carcinoma xenograft tumors had significantly fewer VEGFR2 positive blood vessels than malignant melanoma xenografts.

### Xenograft Responses to Low Dose Metronomic Cyclophosphamide

**Figure 2:**
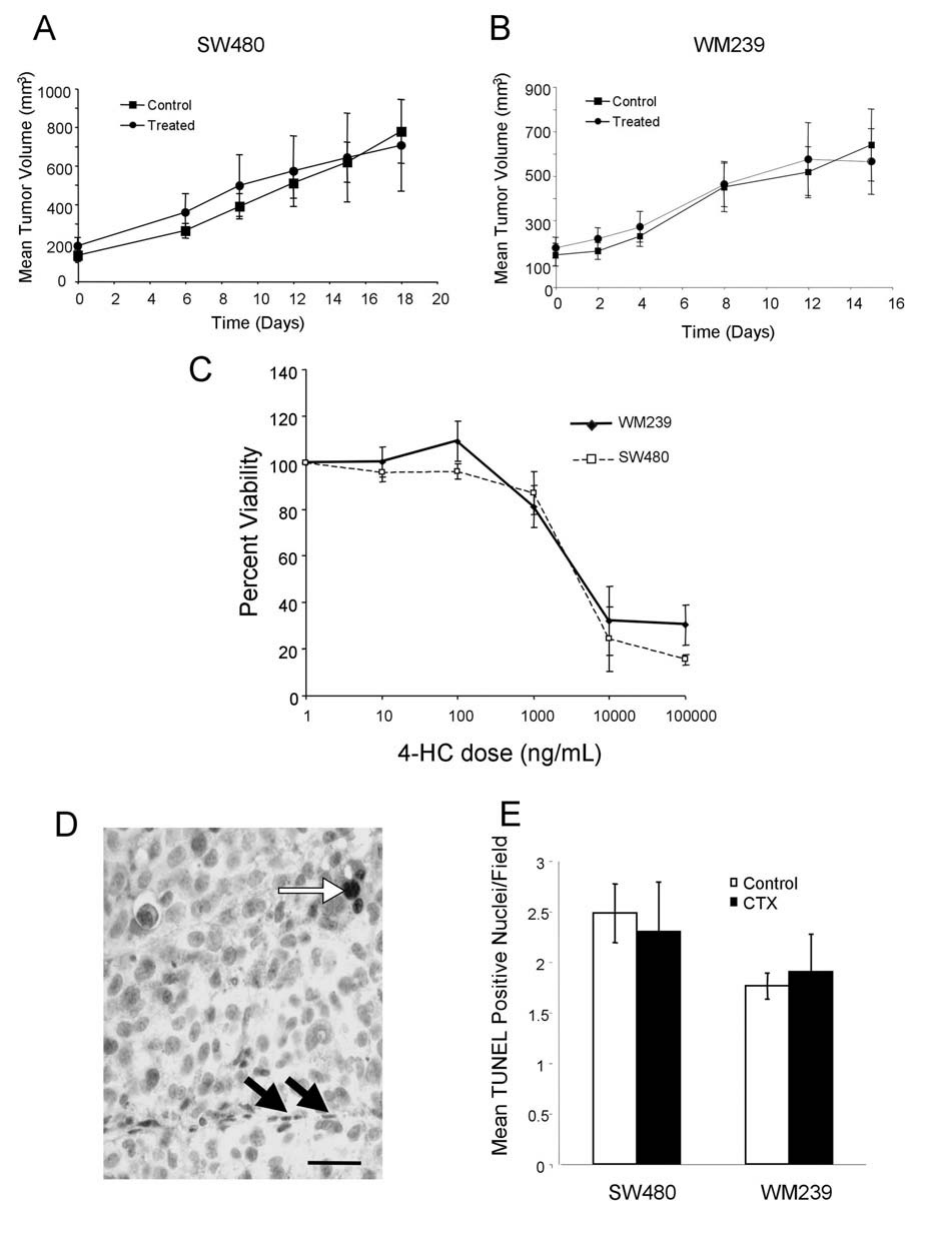
**Mice were treated orally with 20 mg/kg/day of cyclophosphamide in their drinking water**. Tumor growth curves of low dose metronomic cyclophosphamide (CTX) treated and control xenografts of (A) SW480, and (B) WM239 cells; Mean (SEM) is plotted. No significant differences in growth rates were observed between control and treated mice for either SW480 or WM239 xenografts. Cancer cell toxicity to CTX was evaluated *in vitro *using the MTT assay (C). Apoptosis was quantified in tumor sections by TUNEL reaction, and while rare TUNEL positive nuclei were seen in cancer cells (white arrow in D), we did not find any TUNEL positive endothelial cells (black arrows), nor any significant differences in apoptotic cells between control and CTX tumors (E). Scale bar = 25 μm.

**Figure 3:**
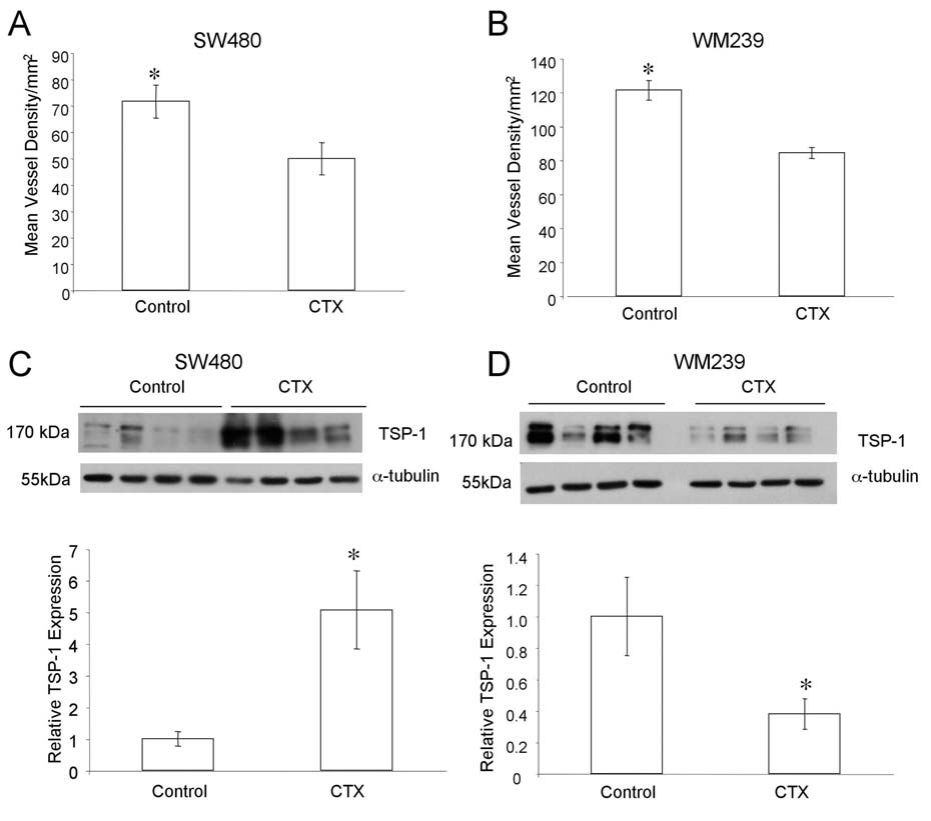
**Microvessel density (MVD) as CD31 positive profiles in SW480 and WM239 subcutaneous xenografts; Mean (SEM) are plotted**. There were significant reductions in MVD in both SW480 (A) and WM239 (B) xenografts between CTX and control groups (*p < 0.05). Western blotting of tumor lysates for TSP-1 revealed significant increases in TSP-1 levels in CTX treated SW480 tumors compared to control (C; *p < 0.05), but significant decreases in CTX treated WM239 tumors compared to control (D; *p < 0.05).

To study the influence of this VEGFR2 heterogeneity on response to low dose metronomic cyclophosphamide therapy (CTX), we evaluated xenografts from treated mice. No significant differences in tumor volume between treated and control groups were observed for either SW480 or WM239 xenografts (Figure 2A). Cancer cell toxicity to CTX was evaluated *in vitro *(Figure 2C), and the approximate LC_50 _of 3000 ng/ml is well above the expected tissue levels of CTX under this metronomic schedule[16]. Consistent with this we found no significant difference in the incidence of cancer cell apoptosis as detected by TUNEL positive nuclei (Figure 2D). Sections of tumors immunostained for CD31 were then scored for microvessel density (MVD). CTX therapy induced a significant reduction in MVD in both SW480 and WM239 xenografts (p < 0.05; Figure 3A). CTX treatment also induced a significant increase in the levels of endogenous anti-angiogenic protein thrombospondin 1 (TSP-1) in SW480 xenografts, but a significant decrease in TSP-1 levels was seen in WM239 tumors treated with CTX compared to control (p < 0.05; Figure 3C).

### Influence of CTX on VEGFR2 Vascular Status

**Figure 4:**
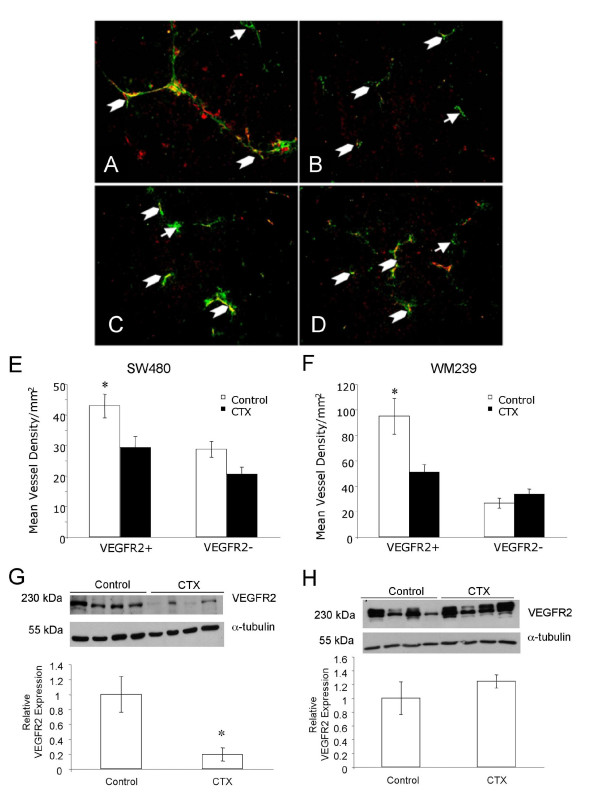
**Effect of low dose metronomic cyclophosphamide therapy on vascular VEGFR2 status**. Examples of VEGFR2 positive blood vessels (chevrons) and VEGFR2 negative blood vessels (arrows) are shown in WM239 (A) treated and (B) control and SW480 (C) treated and (D) control subcutaneous xenografts. Scale bar = 50 μm. Quantification of immunofluorescence showed significant decreases in the density of VEGFR2 positive vessels in CTX treated SW480 (E) and WM239 (F) tumors (*p < 0.05). Total VEGFR2 protein levels in tissue lysates were significantly reduced in SW480 tumors treated with CTX (*p < 0.05), but not in treated WM239 tumors (G, H).

The effect of low dose metronomic cyclophosphamide therapy on VEGFR2 expression was also characterized (Figure 4A-D). There was a significant decrease in mean density of VEGFR2 positive blood vessels between control and treated tumors for both SW480 and WM239 xenografts (p < 0.05; Figure 4E). Western blotting of tumor proteins demonstrated a significant reduction in total VEGFR2 between SW480 control and CTX tumors (p < 0.05), but no significant changes in total VEGFR2 in WM239 (p < 0.05; Figure 4G).

### Hypoxia in CTX and Control Tumors

**Figure 5:**
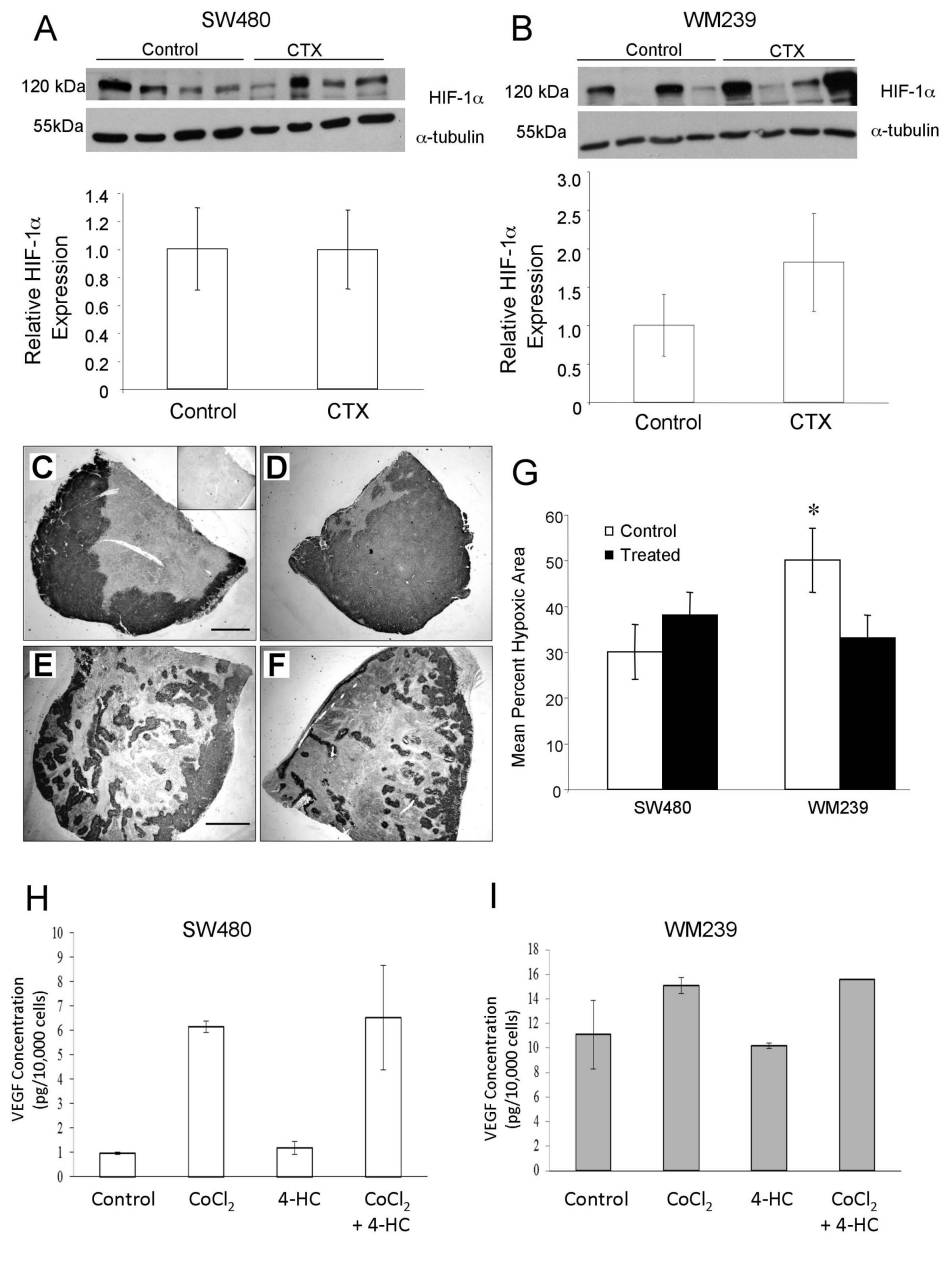
**Effect of low dose metronomic cyclophosphamide therapy on tumor hypoxia**. HIF1-α levels in tumor lysates were not significantly different between CTX and control tumors for either SW480 (A) or WM239 (B). Representative images of tumor cross sections immunostained for CA-IX (dark reaction product) and counterstained with hematoxylin: WM239 CTX (C) and control (D); SW480 CTX (E) and control (F). Insert of panel (C) is negative control without primary antibody. Scale bars = 300 μm. G) Quantification of hypoxic regions (Mean; SEM) in SW480 and WM239 xenografts from CTX and control mice. Treated WM239 xenograft tumors had significantly lower average percent hypoxic regions than control (*p < 0.05). No significant difference was observed between CA-IX staining in SW480 CTX and control tumors. **H, I) **Measurement of conditioned medium by ELISA demonstrates that 24 hour treatment *in vitro *with CoCl_2 _induced up-regulation in VEGF expression in both cell lines (H; SW480; I, WM239), while treatment with low dose 4-HC does not. Combining these treatments had no additive effect over the induction produced by CoCl_2 _alone.

We expected to see that successful angiogenic blockade of SW480 and WM239 xenografts would result in increased tumor hypoxia. However, we found no significant differences in HIF1-α levels between control and CTX for either tumor type (Figure 5A), although the average hypoxic area as demonstrated by CA-IX immunostaining was significantly reduced in WM239 CTX compared to control (p < 0.05; Figure 5C-G). While in vitro induction of HIF1α in WM239 and SW480 cells (by CoCl_2 _exposure) lead to expected increases in VEGF-A production, no significant difference were observed between cells co-incubated with or without CTX (Figure 5H) hence difference in response to CTX between cell lines is not due to differential regulation of VEGF-A expression.

### Blood Vessel Alterations in Response to CTX

**Figure 6:**
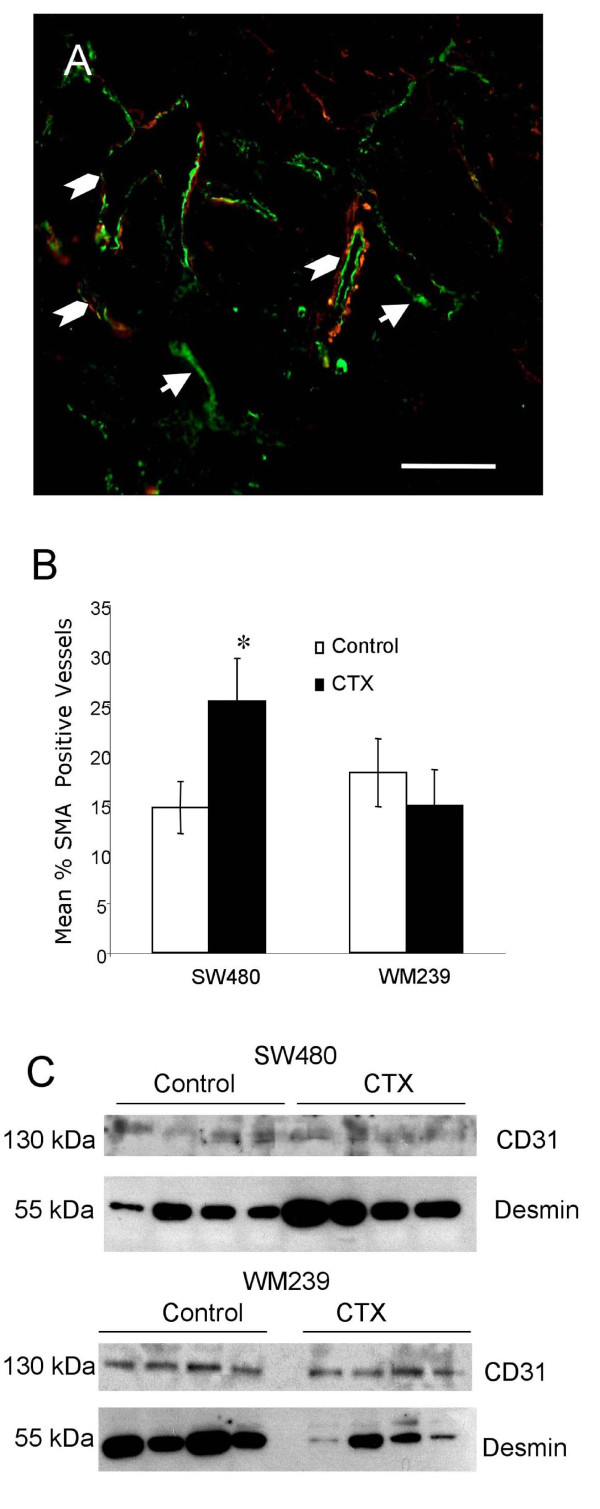
**Effect of low dose metronomic cyclophosphamide therapy on vascular mural cell recruitment**. A) Dual immunofluorescent staining for vascular alpha smooth muscle actin (α-SMA; red) and CD31 (green) showing blood vessels positive (chevrons) and negative (arrows) for this mural cell marker. Scale bar = 50 μm. B) CTX treated SW480 xenograft tumors had significantly increased proportion of α-SMA positive blood vessels compared to control (*p < 0.05). No significant difference was observed between CTX and control WM239 tumors. The ratio between the mural cell marker protein desmin and pan endothelial marker CD31 was assessed by western blotting of whole tumor lysates (C) LDM CTX treatment resulted in increased desmin content realtive to CD31 for SW480 but not WM239 xenografts.

Our finding of reduced MVD but no increase in tumor hypoxia suggests that tumor vessel functionality (*i.e*. perfusion) may have been improved in CTX treated tumors. We found significant increases in the proportion of α-SMA positive (*i.e*. "mature") vessels in SW480 tumors (p < 0.05; Figure 6A). There was also evidence that the relative expression of the mural cell protein desmin was increased in SW480 tumors treated with CTX compared to control but not in WM239 xenografts (Figure 6).

## Discussion

Targeting tumor vasculature as a cancer therapy is now an established concept with the potential to benefit patients with a wide variety of tumor types. However, despite success in preclinical rodent studies this approach had variable results in patients [17-23]. It is now apparent that there are degrees of complexity in the anti-angiogenic process, that the response to its blockade is still not well understood and that cancer cell specific factors influence tumor angiogenesis in unexpected ways [24,25]. For instance, 'resistance' to anti-angiogenic therapy could arise due to redundancy of angiogenic factors, intrinsic or induced co-option of surrounding vasculature, preferential recruitment of bone marrow derived cells that facilitate vascular survival, *etc*.[26,27]. We have previously demonstrated that heterogeneity of endothelial cell Tie2 expression influences anti-angiogenic responses [15]. Here, we explore the impact of VEGFR2 expression patterns in vasculature of colorectal carcinoma (CRC) and malignant melanoma on responses to angiogenic blockade induced by low dose metronomic (LDM) cyclophosphamide.

LDM scheduling of standard cytotoxic agents exerts its therapeutic effect by targeting the endothelial cells of the tumor vasculature, rather than the rapidly dividing cancer cells [4,28-30]. LDM cyclophosphamide (CTX) may exert its anti-angiogenic effect through the up-regulation of TSP-1, shown to target the VEGF/VEGFR2 signaling axis by binding and displacing VEGF [4,6,31-33]. Since SW480 CRC xenografts contained significantly lower proportions of VEGFR2 positive vessels (compared to malignant melanoma), we predicted that superior anti-tumor responses to LMD CTX would occur in WM239 malignant melanoma xenografts. However, despite effective angiogenic blockade in both tumor types, no decreases in tumor volume were seen. Increased progression of experimental tumors during LDM chemotherapy has previously been reported [29,30,34,35], and recent studies indicate that VEGF signaling blockade can also increase cancer cell invasiveness and metastasis [24,25]. Interestingly, although melanoma and CRC xenografts showed significant decreases in MVD in our study, only CRC demonstrated concomitant increases in TSP-1 expression. This suggests that response to LDM CTX is cancer cell specific.

The higher percentage of VEGFR2 positive blood vessels observed here in malignant melanoma may be indicative of greater reliance on VEGF signaling for tumor angiogenesis in this cancer type. Circulating serum levels of VEGF are significantly higher in melanoma patients compared to controls [36], and VEGF-TRAP induced robust anti-vascular and anti-tumor effects in xenografted human melanoma [37]. In fact, WM239 cells themselves express VEGFR2 [Additional file 1: Supplemental Figure S1], thus VEGFR2 detected in tumor lysates is of dual cell origin, which probably accounts for the lack of significant change in VEGFR2 levels overall, despite significant alterations in the proportion of VEGFR2 positive vessels. Tip cells at the leading edge of vascular sprouts express low levels of the Notch-1 and -4 receptors, high levels of their ligand Delta like 4 (Dll4), and low levels of their ligand Jagged, while stalk cells, which form the bulk of the growing sprout are low in Dll4, low in Notch, and high in Jagged[38-40]. This system is thought to function by differential repression (via Notch/Dll4) or stimulation (via Notch/Jagged) of development of tip cell phenotype and hence formation of sprouts[40]. Interestingly, Dll4 signaling through Notch-1 or -4 leads to reduced expression of VEGFR2 in stalk cells[38,41], thus the VEGFR2 free vascular profiles we report here in CRC may represent the stalks of sprouting microcirculation.

Indirectly targeting VEGF in murine xenografts may lead to blood vessel normalization [6,42]. When tumor vasculature is normalized, there is pruning of superfluous 'immature' vessel sprouts, modulation of the pathologically thick basement membrane via activation of matrix metalloproteinases, and increased pericyte coverage mediated by the upregulation of angiopoietin-1[42]. These morphological changes are accompanied by functional alterations such as decreased interstitial fluid pressure, increased tumor oxygenation and improved drug penetration into tumors[11,13,43-46]. In our study, we did not see clear-cut evidence for vascular normalization. While LDM CTX induced several morphological features consistent with enhanced vessel stabilization (increased desmin expression and α-SMA positive mural cell recruitment) in CRC, this did not occur in malignant melanoma tumors despite significant angiogenic blockade in both systems. The lack of increase in TSP-1 production by WM239 cells in response to LDM CTX may have led to altered VEGF signaling in tumor endothelial cells, and hence no changes in mural cell recruitment or vessel stability in these xenografts.

Tumor blood vessels are abnormal in function and structure [10,47,48], leading to regions of transient and chronic ischemia within solid tumors including our CRC and melanoma xenografts. Since microvessel density was significantly decreased in treated WM239 tumors, we expected to see concomitant increased tumor hypoxia [49]. Instead, hypoxic regions decreased, but in the absence of detectable vessel "normalization", suggesting that vessel responses to LDM CTX in melanoma may differ from that of carcinomas. Vessel normalization is transient[42] and the time frame and kinetics of vessel normalization in malignant melanoma has yet to be determined. Further studies are required to determine whether blood flow is increased in the remaining melanoma vasculature after LDM CTX induced angiogenic blockade, which would account for reduced regions of tumor hypoxia.

Most studies of experimental anti-angiogenic treatment report reduced vessel density with parallel reductions in tumor volume, which was not seen here for either cell line. Our previous work demonstrated that WM239 cells can develop a reduced requirement for vascular dependence and hence enhanced survival despite reduced vessel density[50], which may account for the lack of tumor shrinkage seen here. It has also been well documented that ischemic conditions within solid tumors can lead to genetic instability and subsequently tumor progression[51,52]. We have previously shown that 48 hrs exposure to ischemia in vitro could induce de novo *KRAS *mutations in human CRC cells[53]. Accelerated CRC tumor progression due to *KRAS *mutation was also induced in LDM CTX treated xenografts despite a significant anti-angiogenic effect[35]. A similar event whereby increased ischemia leads to development of additional mutations driving tumor progression may be occurring in SW480 tumors here.

## Conclusions

Based on the variable results observed clinically when targeting angiogenesis to treat cancer, we hypothesized that heterogeneous VEGFR2 expression could be responsible for some of the therapeutic outcomes reported to date[54]. Here we demonstrate significant heterogeneity in endothelial VEGFR2 expression in human colorectal carcinoma and CRC xenografts. Despite this, there was robust anti-angiogenic response to metronomic cyclophosphamide chemotherapy, thought to indirectly target the VEGF/VEGFR2 pathway via TSP-1 upregulation. WM239 malignant melanoma xenografts, which display more consistent VEGFR2 vascular expression, also underwent significant reductions in vascular density despite no appreciable change in TSP-1 production. Our findings thus indicate that responses to LDM anti-angiogenic therapy may be highly cancer cell dependent. Further studies are required to determine whether endothelial VEGFR2 heterogeneity, rather than the cancer cell response *per se*, is mediating anti-angiogenic effects in LDM therapy. The mechanisms controlling heterogeneous VEGFR2 expression patterns in different types of cancer also call for investigation, as these potentially could impact responses to targeted anti-angiogenic approaches in a cancer specific fashion.

## Competing interests

The authors declare that they have no competing interests

## Authors' contributions

SGP carried out the xenograft portion of this study, performed immunostaining and data analysis, and drafted the manuscript; UA and KS performed western blotting, ELISA and data analysis; KL performed western blotting and cytotoxic analysis; KM performed α-sma and CAIX analysis; BLC participated in study design and coordination and helped to draft the manuscript. All authors assisted with manuscript preparation and read and approved the final manuscript

## Pre-publication history

The pre-publication history for this paper can be accessed here:

http://www.biomedcentral.com/1471-2407/10/683/prepub

## Supplementary Material

Additional file 1 **Supplemental Figure S1**. Analysis of VEGFR2 expression by WM239 malignant melanoma cells Click here for file
