# Supplementary figures and images for: VEGFR2 heterogeneity and response to anti-angiogenic low dose metronomic cyclophosphamide treatment
Source: BMC Cancer. 2010 Dec 15;10:683. doi: 10.1186/1471-2407-10-683 (PMC3009683; doi:10.1186/1471-2407-10-683)

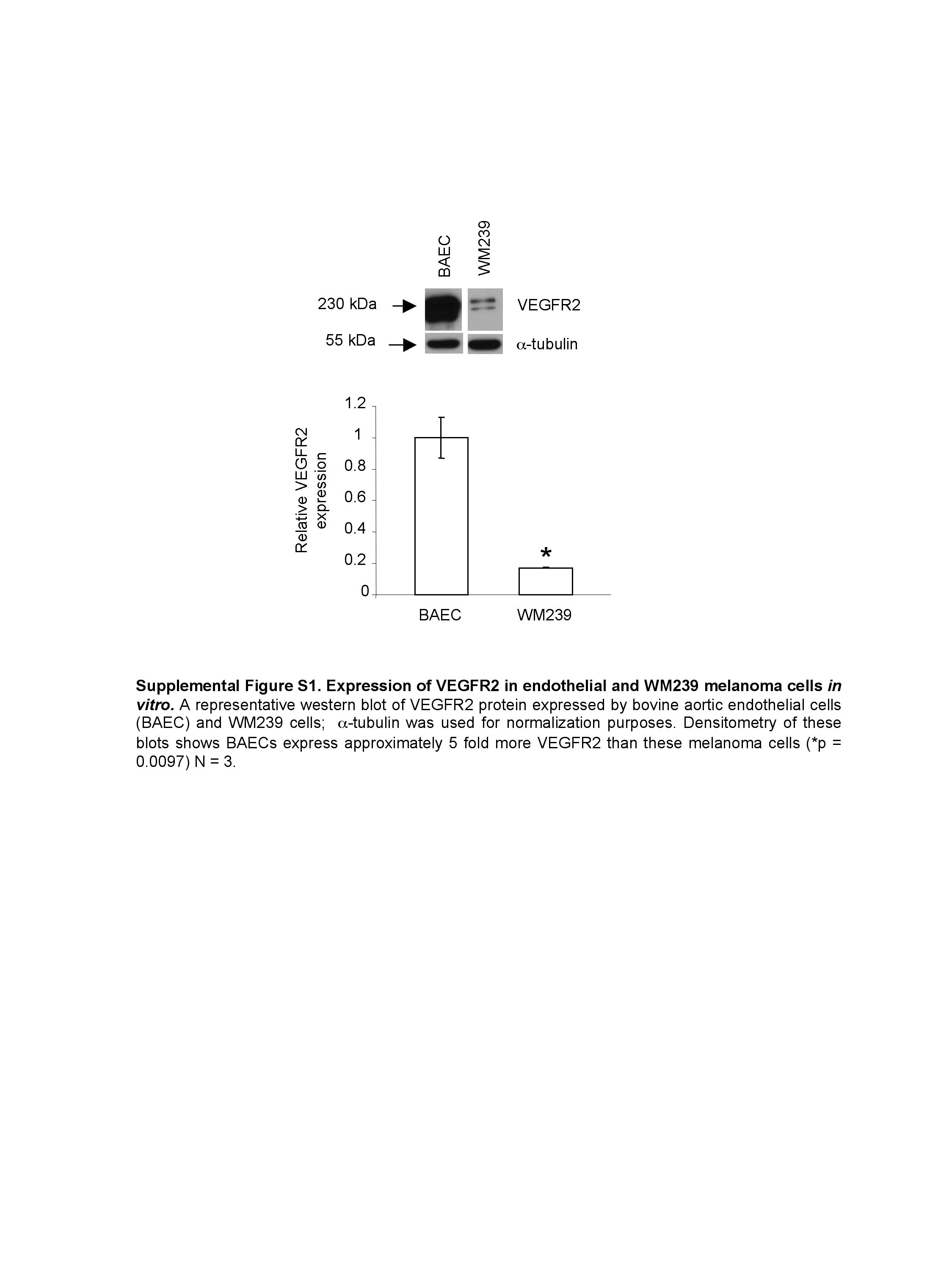

Supplement: Additional file 1 — Supplemental Figure S1. Analysis of VEGFR2 expression by WM239 malignant melanoma cells [file 1471-2407-10-683-S1.JPEG]
